# Supplementary material for: A high-resolution mRNA expression time course of embryonic development in zebrafish
Source: eLife. 2017 Nov 16;6:e30860. doi: 10.7554/eLife.30860 (PMC5690287; doi:10.7554/eLife.30860)
Supplement: Supplementary file 6. [file elife-30860-supp6.zip › biolayout-clusters-files/Cluster156-genes.html]

Cluster156


# Cluster156: Genes

| Ensembl ID | Gene Name | Chr | Start | End | Biotype |
| --- | --- | --- | --- | --- | --- |
| ENSDARG00000101785 | ENSDARG00000101785 | 19 | 43701895 | 43713994 | protein\_coding |
| ENSDARG00000059719 | fam169aa | 5 | 50432911 | 50449986 | protein\_coding |
| ENSDARG00000021366 | fbp1a | 5 | 44174925 | 44243585 | protein\_coding |
| ENSDARG00000019316 | fer1l6 | 9 | 14073107 | 14115839 | protein\_coding |
| ENSDARG00000058603 | jph1a | 24 | 14479412 | 14567178 | protein\_coding |
| ENSDARG00000104839 | mansc1 | 4 | 196259 | 202858 | protein\_coding |
| ENSDARG00000058848 | mcoln1b | 3 | 6105531 | 6149354 | protein\_coding |
| ENSDARG00000069377 | si:dkey-242g16.2 | 10 | 18029854 | 18031345 | protein\_coding |
| ENSDARG00000078250 | zgc:194398 | 5 | 50455417 | 50458078 | protein\_coding |
